# Microscopic Organisms and Antisepsis

**Published:** 1883-02

**Authors:** 


Microscopic Organisms and Antisepsis.—Theories rise and fall, and in the medical world are as warmly espoused or rejected as are polititical tenets in war times.

Years ago the "gaseous theory" of disease was promulgated. To-day its precise import and scope are scarcely known, so completely has it fallen its desuetude. At the present time it is "germ," "bacteria" or "bacillus" on every side. Even the daily papers take occasion to throw gloom over us on the occurrence of a snow-storm; for, they say, the snow covers the hibernating (microscopic) germs of disease, which lurk therein only to come forth with renewed vigor in the spring—and filth—to insidiously kill what children or adult weaklings may happen to be near them.

The bacillus now is on the *tapis*. Is it or is it not? That is the question. Or, given its existence; required, its importance. Some call it a fat crystal. Others, acknowledging that it is an entity and that it is found in phthisical developments, whether catarrhal or tubercular, claim that as it is found in other conditions and not *always* in tuberculosis, mnch if not all of its etiological potency is gone.

The fact that heredity is a great factor in phthisis is universally acknowledged. How can we, then, explain the far-reaching influence of inherited tendencies if a derminate organism is the sole essence of it? No one gees so far as to say that the bacillus is transmitted from generation to generation of human beings, so it is difficult to make the two statements logically agree. Any theory that attempts to state the discovery of the ultimate, essential cause of a disease is apt to be pretty thoroughly riddled before acceptance. And who can point to *one* that has been accepted? What is gout, is a question as far from being answered to-day as it was fifty years ago; although the discovery of the blood condition in gout was thought to herald a new era in pathology and pathogenesis. So with rheumatism, in spite of the fact that lactic acid's association therewith led to impulsive statements about "ultimate causes." One of the most eminent pathologists in the country remarked, a few eays ago, that we are as "far from solving the problem of the cause and essence of disease as were our predecessors of fifty years ago."

Look for a moment at the arguments and dogmatic statements upon which the bacterian and bacilli theories rest. Concerning the former, we know it is found on the diphtheritic membrane in large numbers; but it does seem more likely to be the result of decay of tissue than the cause thereof. For after gangrenous cavities are formed in the lung, following compound fractures of the ribs, as from the kick of a horse, the bacteria exist in the putrid fluid in very swarms. Now, the cause of the fracture and subsequent gangrene did not depend on *minute* organisms, surely.

Then, again, one coterie of observers cultivate the bacteria, inject them into the blood of animals, and directly there follows a special and peculiar lésion—tuberculosis, as a rule, is the malady concerning which the experiments are made.

Others go through the same operation, step by step, and arrive at negative results only.

Now, upon the view that each man holds regarding germs, whether atmospheric or no, will depend in very great measure his opinion and employment of vigorous and elaborate antisepsis.

At this day, many thoughtful surgeons are abandoning Listerism. Lanson Tait performed 100 consecutive ovariotomies with but three deaths, and in *none* was the Listerian method employed. That is to say, they regard the cleanliness made necessary by Prof. Lister's method as its sole good point, and look upon the elaborate details of its application as worthless. Carbolic acid is no longer a panacea. Some wounds will do well under the most careless treatment; others, in spite of the most careful dressing, after Lister's method, will exhibit signs of gangrene, will be covered with ichorous pus, and will be accompanied by pyæmia. We know of an example in point: in one of the largest hospitals in America the Listerian method was employed in *but one* in sixteen amputations. In this one *only* did gangrene occur. This is not proof, of course, but 'tis enough; it will serve to show that antisepsis is not the greatest discovery of modern surgery. Finally, one can not help noticing that as the claim of bacteria and germs being the causes of everything, even lobar pneumonia, has been slowly relinquished, so the advocates of Listerism and antisepsis have been less strenuous in their efforts to prove that a certain routine must in every case be observed if recovery is to be expected or complications warded off.

